# First molecular detection of hepatitis E virus genome in camel and pig faecal samples in Ethiopia

**DOI:** 10.1186/s12985-021-01626-9

**Published:** 2021-08-04

**Authors:** Fufa Dawo Bari, Haimanot Belete Wodaje, Umer Said, Hika Waktole, Melaku Sombo, Samson Leta, Tesfaye Rufael Chibsa, Paul Plummer

**Affiliations:** 1 Department of Microbiology, Immunology and Veterinary Public Health, College of Veterinary Medicine and Agriculture, Addis Ababa University, Bishoftu, Ethiopia; 2 National Animal Health Diagnostic and Investigation Center, Sebeta, Ethiopia; 3 Department of Biomedical Sciences, College of Veterinary Medicine and Agriculture, Addis Ababa University, Bishoftu, Ethiopia; 4 Department of Veterinary Diagnostic and Production Animal Medicine, College of Veterinary Medicine, Iowa State University, Ames, IA USA; 5 Department of Microbiology and Preventive Medicine, College of Veterinary Medicine, Iowa State University, Ames, IA USA; 6 Present Address: Assosa University, Assosa, Ethiopia; 7 Present Address: Oda Bultum University, West Hararge, Chiro, Ethiopia

**Keywords:** Dromedary camels, Hepatitis E virus, Nested RT-PCR, Pigs, Ethiopia

## Abstract

**Background:**

Hepatitis E is an enteric and zoonotic disease caused by hepatitis E virus (HEV) that is mainly transmitted via the faecal-oral route through contaminated food or the environment. The virus is an emerging infectious agent causing acute human infection worldwide. A high seroprevalence of the disease was reported in pregnant women in Addis Ababa, Ethiopia, raising significant public health concern. The presence of HEV specific antibodies were also reported in dromedary camels in the country; however, the infectious virus and/or the viral genome have not been demonstrated to date in animal samples.

**Methods:**

To address this gap, a total of 95 faecal samples collected from both apparently healthy pigs of uncharacterised types (50 samples) in Burayu and Addis Ababa areas and camels (*Camelus dromedarius*, 45 samples) in west Hararghe were screened for the presence of HEV genome using universal primers in a fully nested reverse transcription polymerase chain reaction (nRT-PCR). The protocol is capable of detecting HEV in faecal samples from both pigs and camels.

**Results:**

The nRT-PCR detected HEV genes in six (12%) pig faecal samples and one camel sample (2.2%). Therefore, the results indicate that HEV is circulating in both pigs and camels in Ethiopia and these animals and their products could serve as a potential source of infection for humans.

**Conclusion:**

The detection of HEV in both animals could raise another concern regarding its public health importance as both animals’ meat and camel milk are consumed in the country. Further studies to determine the prevalence and distribution of the virus in different animals and their products, water bodies, food chain, and vegetables are warranted, along with viral gene sequencing for detailed genetic characterisation of the isolates circulating in the country. This information is critically important to design and institute appropriate control and/or preventive measures.

## Introduction

Hepatitis E is a zoonotic, emerging enteric infectious disease caused by Hepatitis E virus (HEV). The disease is a major cause of water-borne hepatitis epidemic in tropical and subtropical countries with poor sanitary conditions including southeast and central Asia, the Middle East, and Africa [1]. The HEV belongs to the genus *Orthohepevirus* in the *Hepeviridae* family that has four species, *Orthohepevirus* A with eight genotypes in mammals, the *Orthohepevirus* B circulating in chicken, the *Orthohepevirus* C circulating in rats and ferrets, and the *Orthohepevirus* D circulating in bats [2]. The genome of HEV particle is comprised of a positive, single-stranded RNA packed inside the icosahedral capsid proteins [2]. Two major species of the virus are recognised: avian HEV and mammalian HEV. The mammalian HEV is zoonotic and causes disease in human beings while the avian HEV causes enlargement of liver and spleen in chickens, but not in humans. The mammalian HEVs include several genotypes that can infect specific animals differently. HEV genotype 1 (HEV-1) and HEV-2 are human viruses. They are highly endemic in several parts of Asia, Africa, the Middle East, and Mexico. They spread through contamination of water supplies with human faeces i.e. via faecal—oral route due to faecal contamination of drinking water [3]. In contrast, genotypes 3 and 4 are zoonotic and infect humans and several other animal species, such as pigs, wild boar, and deer [4–6]. The zoonotic transmission of genotype 3 or genotype 4 occurs through consumption of undercooked meat or contact with infected animals [7]. Genotype 5 and genotype 6 infect wild boar, while genotype 7 infects camels. A recent study showed zoonotic transmission of the genotype 7 HEV was related to the consumption of camel meat. Genotype 8 infects the Bactrian camel [8]. In general, five genotypes of the *Hepeviridae* family namely genotypes 1–4 and 7 are known to infect humans [9, 10].

Hepatitis E in humans is characterized by large scale water-borne epidemics of jaundice in regions of the world with contaminated water supplies and low sanitary conditions. According to World Health Organisation (WHO) report [11], there are an estimated 20 million HEV infections worldwide, leading to an estimated 3.3 million symptomatic cases of hepatitis E infection. In 2015, hepatitis E caused approximately 44,000 deaths accounting for 3.3% of the mortality due to viral hepatitis [11]. In humans, the disease is generally self-limiting; however, mortality rates are higher among pregnant women and young infants. Chronic HEV infection is a particular problem for immunocompromised patients, such as those who have received a solid organ transplant and those with human immunodeficiency virus infection [12]. In addition to humans, HEV has been found in other mammals: pigs, boar, deer, rodents, ferrets, rabbits, mongoose, bats, cattle, sheep, foxes, minks, and horses [13, 14]. Human infections with HEV genotype 3 (HEV3) and HEV genotype 4 (HEV4) have been associated with consumption of raw or undercooked pork meat [15]. In general HEV infection is mainly transmitted through contaminated water with animal infected faeces including that of dromedary camels [12].

In African countries, a number of HEV outbreaks were reported including in Ethiopia, Somalia, Uganda, Democratic Republic of Congo, Sudan and South Sudan in different periods [16, 17]. The highest seroprevalence (50.01%) was reported in North Africa followed by Ethiopia, East Africa (35%) [18]. In Ethiopia there is a report of high seroprevalence (31.6%) of HEV in pregnant women [19] from a single hospital located in Addis Ababa. In addition, a seroprevalence study of HEV carried out in camels in Ethiopia reported evidence of immune response (antibody detection) to the virus [20]. Here, we report the first molecular detection of the HEV gene in faecal samples of dromedary camels collected from western Hararghe and uncharacterised types of pigs found in farms in and around Addis Ababa, Ethiopia using universal primers and a nest reverse transcription polymerase chain reaction technique.

## Materials and methods

### Study area

The study was conducted from November to April in 2020 in Burayu (Oromia) and in Kolfe Keraniyo, Addis Ababa. Burayu town is located in Oromiya National Regional State and in the western direction of Addis Ababa at a distance of 15 km from the capital city. The town is a highland area located at an altitude of 2580 m above sea level with an area of 66.5 km^2^. Burayu town is bounded by Finfinnee (Addis Ababa) city in the East, Walmera district in West, Sululta district in North and Sebata Hawas district in South [21]. In addition, dromedary camels found in Western Hararghe were included in this study. Western Hararghe is located in Oromia regional state, Eastern part of Ethiopia, at a distance of 317 km away from Addis Ababa [22, 23].

### Study population

Apparently healthy pigs and camels of all age groups and both sexes kept under farmers management conditions were used for the study. The pigs were found in Addis Ababa, Ashewa Meda, and Burayu towns. The exact number of pig farms and pig population in the Burayu district was not known as there is no record of pig farms and population kept by the office of livestock and fisheries of Burayu district. However, in Burayu district and in Kolfe Keraniyo (a sub city in Addis Ababa), there were three and one extensive pig farms, respectively, with herd sizes of 50–100.

The dromedary camels were found in western Hararghe zone of Oromia regional state, Ethiopia that has approximately 91,948 camel populations [22]. Based on proximity and accessibility of the area, two towns namely Chiro and Mieso towns, were selected for the study.

### Study design and sample collection

A cross sectional study design was conducted to select the pigs and camels for faecal sample collection from their respective study areas that is in and around Addis Ababa and western Hararghe, respectively. Four pig farms were included for sample collection. A total of 50 pig faecal samples were collected, of which 41 were directly collected from the pig rectum while 9 samples were collected as freshly voided faeces from the barn. The rectal faeces were collected by inserting two fingers into the rectum when the pigs were in recumbent position and placed in sterile universal tubes. These samples were labeled and transported at 4 °C to the National Animal Health Disease Investigation Centre (NAHDIC) virology laboratory, Sebeta. They were stored at − 80 °C until molecular detection was conducted. A total of 45 camel faecal samples (26 samples were collected from Chiro town and 19 from Mieso town) were collected. In addition, the pig watering area and grazing areas were visited to assess the potential for disseminating the disease to other animals and humans in the area. The camel faecal samples which had been collected in 2019 from western Hararghe were already stored at − 80 °C until the molecular detection commenced. A nearby watering point or river was visited to evaluate the area for contamination with swine faeces. In pigs all sample were collected from females while in camels both female and male were sampled.

### Molecular detection of the HE virus

#### RNA extraction

Total RNA was extracted from the faecal samples using the QIAamp viral RNA extraction mini kit (QIAGEN, Hilden, Germany). Briefly, 10% suspension of faecal samples were first prepared by suspending it in 5 ml of phosphate-buffered saline (PBS) and then centrifuged at 8000 revolution per minute (rpm) for 5 min. The supernatant obtained was collected and used for extraction of viral RNA according to the QIAamp® Viral RNA Mini kit 2018 instructions.

#### Amplification of HEV RNA gene using fully nested RT-PCR

The extracted RNA was amplified with fully nested conventional reverse transcriptase polymerase chain reaction (nRT-PCR) in two consecutive amplification rounds [24]. The RT-PCR targeted to amplify about 469 base pair (bp) nucleotides in the first round and 325 bp in the second round in the 5ʹ end of open reading frame 1 (ORF1). The first round conventional RT-PCR amplification was conducted with forward and reverse external primers (HEV-CS) and (HEV-CAS) with primer set: HEV-CS 5’TCG CGC ATC ACM TTY TTC CAR AA 3’ and HEV-CAS 5’ GCC ATG TTC CAG ACD GTR TTC CA 3’, respectively, followed by the second RT-PCR. The initial RT-PCR was performed with an one-step RT-PCR Kit (Qiagen, Hilden, Germany) in a 25µL volume according to Zaki et al. [24].

The second amplification was performed with internal primers: (HEV-CSN) 5’ TGT TGC CCT GTT TGG CCC CTG GTT TAG 3’ and (HEV-CASN) 5’ CCA GGC TCA CCR GAR TGY TTC TTG CA 3’ in a 50µL reaction mixture volume as described by López-Santaella et al. [25]. A FlexCycler^2^ thermal cycler was used for genome amplification. The amplification product was separated by gel electrophoresis on a 2% agarose gel for 40 min at 100 voltages. SYBR Safe Dye (Invitrogen, Carlsbad, USA) staining was used to reveal the amplification products documented in a gel documentation apparatus. The bands of positive results were aligned at 325 base pair (bp) with 100 bp PCR marker (ladder) (Qiagen, genpilot) and the amplification was gel documented.

### Data management

Age, sex, and location of both pigs and dromedary camels were recorded. Laboratory results was recorded in Microsoft Excel spread sheet for data analysis using proportion method. The RT-PCR results where documented in gel electrophoresis gel documenting apparatus.

## Result

### Samples collected and faecal contamination around watering points

The age distribution of studied pigs range from 3 months to 4 years. Of the 50 pigs sampled, 54% (27/50) faecal samples were collected from females while 28% (14/50) were collected from males. The remaining 18% (9/50) samples were collected from freshly voided faeces from the barns. Pigs feeding in dirty environment around river in Burayu area were found and such feeding habit could further contaminate the river.

### Viral RNA detection

**Fig. 1:**
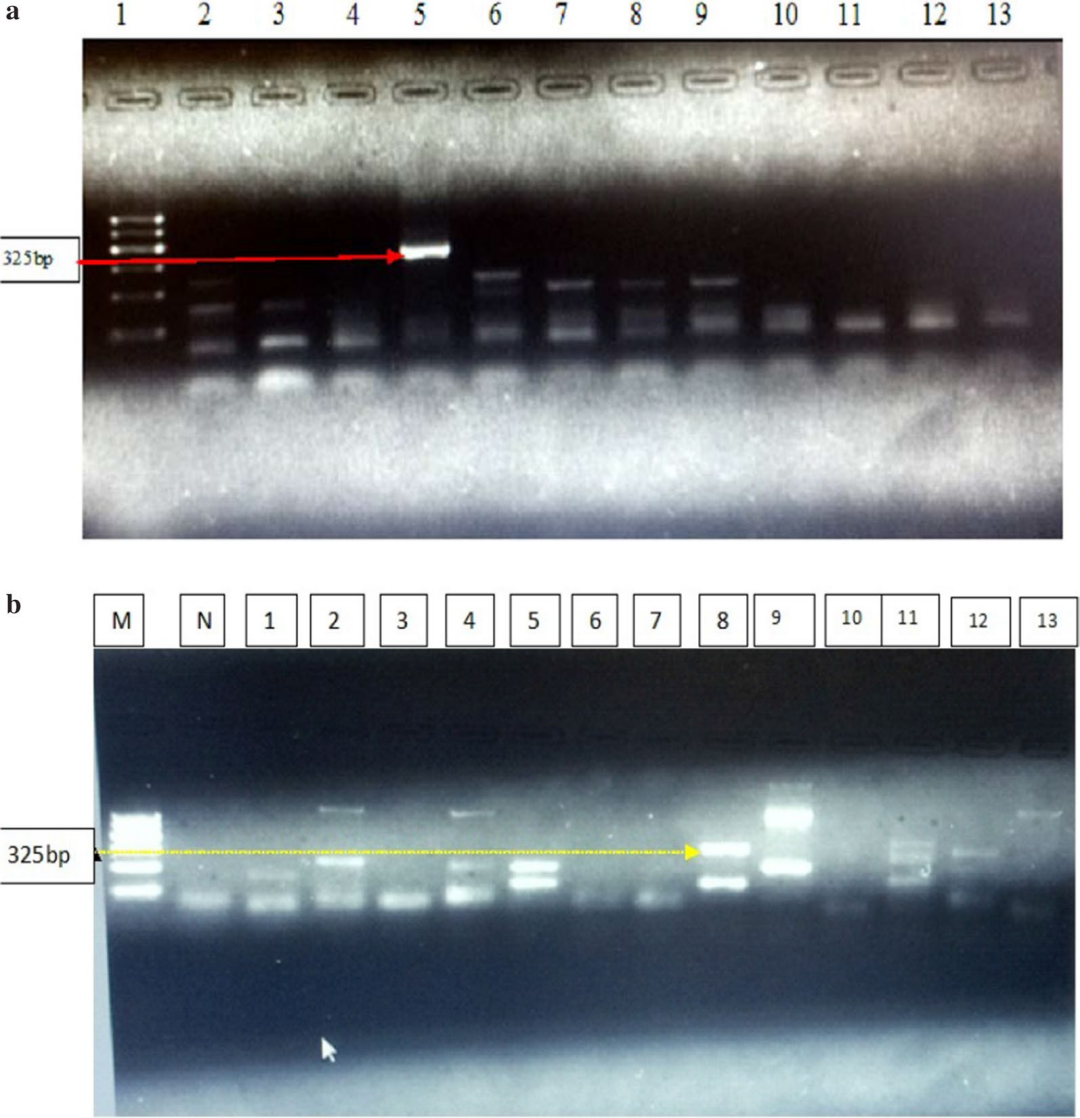
**a** Agarose gel electrophoresis of HEV gene products amplified by nRT-PCR. Lane 1: One kilo bp molecular marker standard. Lane 5 gave expected band size of 325 bp which is strong positive; lanes 2, 3, 4, 6–13 are negative results. **b** M = 1kilo bp DNA molecular markers (ladder), N = negative control, 1–7 camel faecal samples, 8–13 pig faecal samples. Sample 8 gave expected band size of 325 bp. Camel sample 2 was considered positive while samples 4 and 5 were considered as doubtful because they gave band size slightly lower than expected band of 325 bp

Of the total 50 pig samples detected by fully nested RT-PCR, 12% of the collected faecal samples (6/50) gave positive result for HEV viral RNA genome amplification by giving expected band size of 325 bp as visualized on agarose gel electrophoresis (Fig. 1a, b). In camels, 2.2% faecal sample (1/45) was positive (Fig. 1b: lane 2) and 4.4% (2/45) faecal samples (Fig. 1b, lanes 4 and 5) generated band very close to but slightly lower than the expected 325 bp. The other samples from both animal species gave negative results. In these Fig. 1a and b below, lanes 5 and 8, respectively, gave strong band, suggesting clear HEV genome amplification while in two pig samples, light bands of similar band sizes (Fig. 1b, lanes 11 and 12) were observed.

## Discussion

In this study, the 12% molecular detection of HEV genome in faecal samples of domestic pigs and the 2.2% results, in dromedary camels’ (Fig. 1 a, b) was reported in animals for the first time in Ethiopia. Previous serological detection of immunoglobulin G (IgG) of HEV virus in humans [19] and in camels found in Ethiopia [20] and molecular characterisation in human samples [26, 27] were reported. The current result provided strong proof and molecular justification for the detection of the virus genome in pigs and camels faceal samples in their respective study sites in the country. The detection of HEV on pig farms and camels is clear indication of the potential risk of the virus to the public because both pig and camel meat and camel milk are consumed in the capital city Addis Ababa and in camel producing areas. The pigs found around Burayu were between 3 and 4 years of age. These pigs have frequent contact with people and other animal species posing a risk for disease transmission to susceptible hosts. The nested RT-PCR used universal primers that can detect HEV viruses genome and the finding is slightly higher than the 10.1% report from pigs in Ghana [28] but higher than the 5.9% report from Cameroon [29]. In European countries like Serbia and Bulgaria, very high seroprevalence of 34% and 74% HEV in young slaughter pigs [30, 31] were reported. Pigs usually acquire the infection at early age when maternal immune response wans and the prevalence increase with increase in age of pigs. John et al. [9] also reported similar findings in wild rates using the same amplification forward and reverse primers of the first round RT-PCR primers except that the second round primers they used have slight modification. In addition, this study also agreed with Widen et al. [32] report who used the nested RT-PCR for detection and analysis of potentially zoonotic HEV in French rats. In other livestock species and humans, prevalence data and knowledge of circulating genotypes of HEV in the livestock population is not known as there were no former molecular detection and characterisation studies conducted in the country.

Previous reports indicated that HEV viruses in pigs are zoonotic and belong to genotypes 3–6 [2, 8, 33, 34]. In General, many viruses infecting pigs are known to infect humans and has the ability to cross species barriers [35, 36]. In African countries like Nigeria, the widespread distribution of HEV reported with prevalence rate of 76.7% of genotype 3 [37]. All these reports are pointing to increased responsiveness, surveillance, and prevalent nature of this virus in the environment. In Ethiopia there is no serological and molecular evidence of prevalence of HEV in pigs and this report is the first molecular evidence regarding HEV gene detection.

The role of pigs as potential HEV reservoirs is well documented in many parts of the world. For example, the incidence of pig HEV was reported in United Kingdom, China, Japan, and Canada [36, 38, 39]. In Africa, over 20% of pig faecal samples have been confirmed positive for HEV RNA in South Africa 37, and 76.7% of genotype 3 in Nigeria [40]. In pigs, grazing together, rooting, and nosing, as well as wallowing in mud is their natural behaviour. Such pig behaviours are associated with wallowing including feeding, drinking, defecating, and urinating in the mud [41] and water bodies suggests the probable route of contamination of the environment with HEV virus. In Ethiopia, there is no serological and molecular study that report prevalence of HEV in pigs and other animals’ species except in camels.

The 32% serological evidence of HEV infection in pregnant women in a hospital in Addis Ababa [19] suggests the public health threat of the disease. The place from which the women visited the hospital is unknown to link the relationship of the women with pigs found in and around Addis Ababa. But, the pig production system is extensive type in both Burayu and Ashewa Meda areas of Oromiya special zone around Finfinnee (Addis Ababa) and in the city of Addis Ababa. The contamination of soil, watering points and other materials that could expose the virus to susceptible hosts including humans is likely and the observation of high seropositive in pregnant women is expected. In Abebe et al. [19] study, the exact location of the pregnant women other than evidence of detection in hospital settings were not reported to correlate source of acquiring the virus with exposure to any animal species.

In dromedary camels, the detection of HEV genome strengthens previous serological report of anti-HEV IgG antibody in more than 20% of camels found in Afar regional state, Ethiopia [20]. Such serological detection suggests the HEV infection has likely spread among dromedary camels in Ethiopia. The serological report of 20% was from Afar region while the current report is from Oromiya region which are two different regions. From the serologically positive areas of Afar, no further study in dromedary camels were conducted to grow the virus and/or to molecularly detect the virus genome. This report is the only molecular evidence available in the country that could show detection of the virus genome in camel faecal samples. Both the pig and the camel results need further confirmation by gene sequencing and sequence analysis to elucidate the evolutionary relationship and emergence of the virus in current hosts and other host species. Similar serological study conducted in different countries like Kenya, United Arab Emirate, Somalia, Sudan, Pakistan, and Egypt reported detection of 31–63% [42] which is much higher than the report from Ethiopia. The limitation of the current study is both antibody and genome detections were carried not carried out simultaneously from serum and faecal samples collected from the same animal to present the real prevalence of HEV infection and this need to be addressed in the future.

Usually, large numbers of camels and other domestic animals from many different herds/flocks congregate at watering and fresh grazing sites that create a perfect condition for disease transmission and spread among susceptible animals. During milking, washing of hands, milking vessels, the udder and teats are not washed properly by many milkers’ prior to milking the camels. Besides, the milking area is generally full of dust and dung and without shade. These conditions not only affect the quality and safety of the produced milk [43, 44] but also provide good way of HEV contact with susceptible hosts and acquiring of infection [20].

HEV RNA has been detected in diverse food products ranging from meat and seafood, to fruits and vegetables [45]. According to Kokkinos et al. [45, 46] HEV was reported in 5% of the irrigation water samples and 3.2% of fresh lettuce. Raw and undercooked fruits and vegetables are normally sold to the consumer in a ready to eat form in Addis Ababa. Most of vegetation’ in southern direction around Addis Ababa used irrigation water containing sewages released from the city and pig producing farms, which may be the source of vegetable contamination. Therefore, one route of virus transmission to the community in the study area may be through use and/or drinking of contaminated water and consumption of raw vegetables such as cabbage with HEV—contaminated water.

## Conclusion

In this study, molecular detection of the HEV from faecal samples of pigs and from a camel sample using a nested RT-PCR is reported for the first time in Ethiopia. The observation of pigs grazing in the field and around watering points, suggest possible environmental contamination of the virus that could lead to infection of susceptible animal species including humans. The HEV virus is becoming the leading cause of enterically transmitted viral hepatitis in humans [47]. These viruses are transmitted by the faecal-oral route and many of the environmental and socio-economic factors foster the transmission routes. Although, HEV gene is successfully detected in this study, the gene of the viruses were not sequenced to determine their exact genotype. Therefore, further research is recommended to generate HEV gene sequence data so as to determine the genotype/s circulating both pigs and camels together with other domestic animals in the country.

## Data Availability

All data generated or analyzed during this study are included in this article.

## Abbreviations

bp: base pair
cDNA: complementary DNA
DNA: deoxyribonucleic acid
HEV: hepatitis E virus
IgG: immunoglobulin G
Min: minute
NAHDIC: National Animal Health Disease Investigation Centre
nRT-PCR: nested reverse transcription polymerase chain reaction
PBS: phosphate-buffered saline
ORF: open reading frame 1
PCR: polymerase chain reaction
QGIS: quantum geographic information system
RNA: ribonucleic acid
Rpm: revolution per minute
USA: United States of America
WHO: World Health Organisation
